# Supplementary material for: Liquid crystalline collagen assemblies as substrates for directed alignment of human Schwann cells
Source: Soft Matter. 2024 Nov 4;20(45):8997–9006. doi: 10.1039/d4sm00534a (PMC11533399; doi:10.1039/d4sm00534a)
Supplement: SM-020-D4SM00534A-s001 [file SM-020-D4SM00534A-s001.pdf]

Supplementary Information (SI) for Soft Matter.
This journal is © The Royal Society of Chemistry 2024

Supplementary Information for:

Liquid Crystalline Collagen Assemblies as Substrates for Directed Alignment of Human Schwann Cells

The morphological characteristics of cholesteric bands, including parameters such as the width (representing the width of the twisting fibrils in each individual cholesteric bands; designated as w), the spacing (representing the distance between adjacent cholesteric bands; notated as s), and the diameter of the birefringent cord (representing the diameter of the birefringent cholesteric bands; denoted as d), were quantitatively analyzed using ImageJ software (refer to Figure S1).

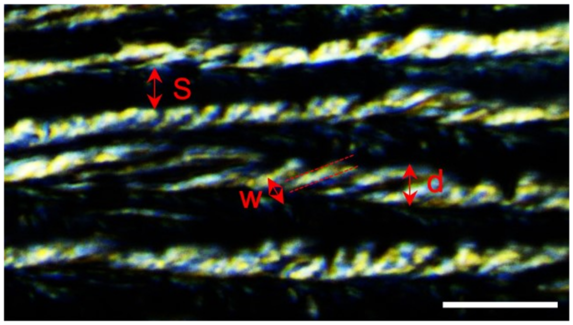

Figure S1. Depiction of the analyzed parameters, including width (w), spacing (s), and birefringent cord diameter (d), in the quantitative analysis of cholesteric bands' morphology. Scale bar represents 15 µm.

The statistical distributions of these parameters are displayed through histograms in Figure S2A-C. The order parameter (S) of the cholesteric bands was determined by evaluating the alignment of individual cholesteric bands in relation to the director.

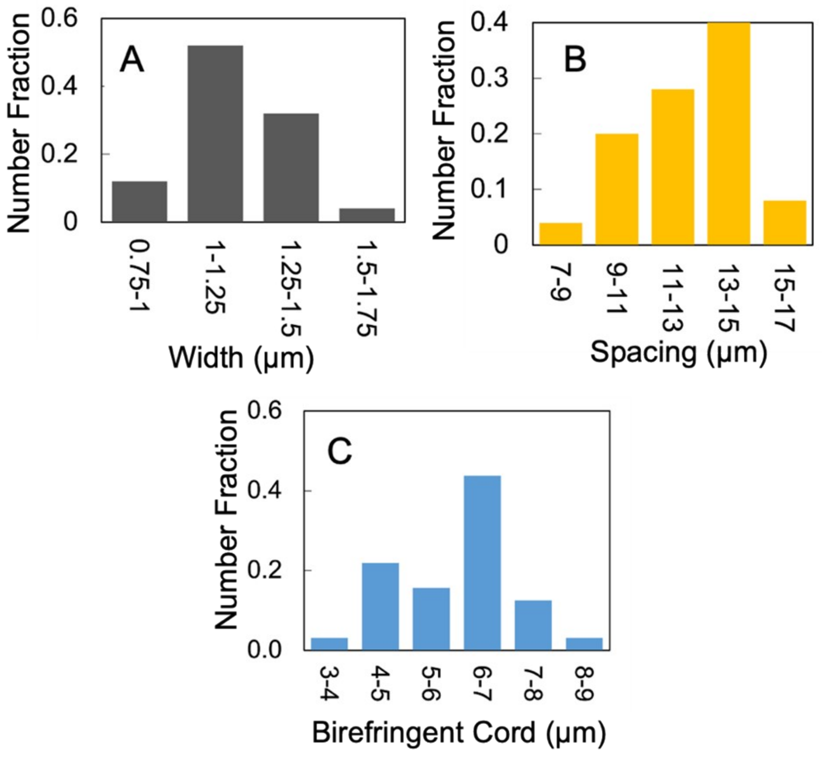

Figure S2. Characterization of cholesteric bands in LC collagen film: (A), (B) and (C) Histograms depicting the distribution of number fractions with respect to the width, spacing, and thickness of the birefringent cord, respectively.

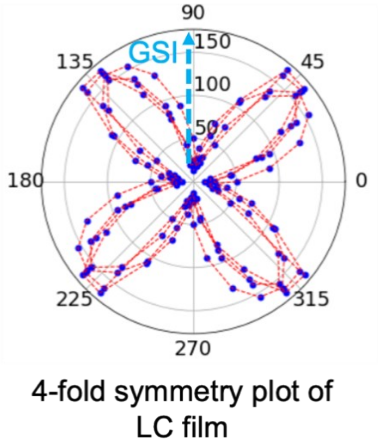

Figure S3. Polar plot of gray scale intensity as a function of rotation angle (°), generated from a sequence of images taken from LC collagen film under cross-polarized light while rotating the microscope stage in 10° increments. The radial axis represents the gray scale intensity (GSI).

The complete extinction of light under the cross-polarized optical microscope for control collagen hydrogel in Figure S4 demonstrates the isotropic structure of collagen.

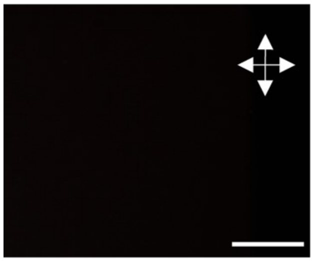

Figure S4. Cross-polarized optical micrograph illustrating a control collagen hydrogel. This hydrogel, derived from the same collagen precursor solution, was prepared in a 6-well plate on bare microscope glass without exposure to a magnetic field, allowing the formation of a gel structure. This control serves as a baseline for assessing the impact of a magnetic field on collagen alignment. The scale bar represents 100 μm.

Figure S5 compares the morphology of cells cultured on the isotropic control and LC hydrogel samples, focusing on those that do not adopt the typical bipolar spindle-like shape, which is characteristic of cells lying flat in the plane of observation. On the isotropic hydrogels, approximately 75% of the cells exhibit non-parallel orientations, appearing as either nearly circular (highlighted in red circles) or triangular (highlighted in yellow circles). The circular cells are likely those oriented perpendicular to the plane, while the triangular cells are positioned at intermediate angles, neither parallel nor fully perpendicular to the surface. In contrast, only 10% of the cells on the LC hydrogel deviate from the typical flat, spindle-like morphology, indicating that the unique LC structure promotes a more uniform alignment of cells parallel to the surface. This observation aligns with theoretical predictions based on the probability of cells adopting different orientations in a random space, such as the control hydrogel, where there is no inherent preference for alignment.

Orientation Distribution Analysis: Cells within the hydrogel can theoretically adopt any orientation in 3D space. To quantify the fraction of cells lying flat, angled, or perpendicular, we need to consider spherical coordinates, where orientations can be described using two angles:

Theta (θ): The angle between the cell's major axis and the hydrogel surface, ranging from 0° to 180°.

Phi (φ): The azimuthal angle, ranging from 0° to 360°.

Based on these angles, the orientations can be categorized as follows:

Flat cells: Defined as having their major axis parallel to the hydrogel surface, corresponding to θ values near 0° or 180°.

Perpendicular cells: These would correspond to θ values near 90°, making them perpendicular to the hydrogel surface.

Angled cells: All other θ values represent cells at various angles relative to the surface.

Calculating the Probability for Flat Cells: If we assume a random distribution of cell orientations within the hydrogel, the fraction of cells that are flat can be determined by considering the area subtended by θ values close to 0° and 180° on a unit sphere. For simplicity, we define "flat" cells as those with θ angles between 0°–15° and 165°–180° (i.e., within 15° of being perfectly flat). The remaining orientations would be considered non-flat.

The surface area of a unit sphere is proportional to the integral of the solid angle, which depends on θ. For a uniform distribution, the probability P(flat) is given by the fraction of the total solid angle:

$$P(\text{flat}) = \frac{\int_0^{15} \sin\theta \, d\theta + \int_{165}^{180} \sin\theta \, d\theta}{\int_0^{180} \sin\theta \, d\theta}$$

$$\int_0^{15} \sin\theta \, d\theta = \cos 0^\circ - \cos 15^\circ \approx 1 - 0.9659 \approx 0.0341$$

$$\int_{165}^{180} \sin\theta \, d\theta = \cos 165^\circ - \cos 180^\circ \approx -0.9659 - (-1) \approx 0.0341$$

The total probability for flat orientations:

$$P(\text{flat}) \approx 0.0341 + 0.0341 = 0.0682 \approx 6.8\%$$

Since the remaining orientations are considered non-flat (angled or perpendicular):

$$P(\text{non-flat}) = 1 - P(\text{flat}) = 1 - 0.0682 = 0.9318 \approx 93.2\%$$

We posit that edge effects and finite thickness of the hydrogels account for the observed 75% of cells with non-parallel orientations in control samples, in contrast to the predicted value of 93.2%.

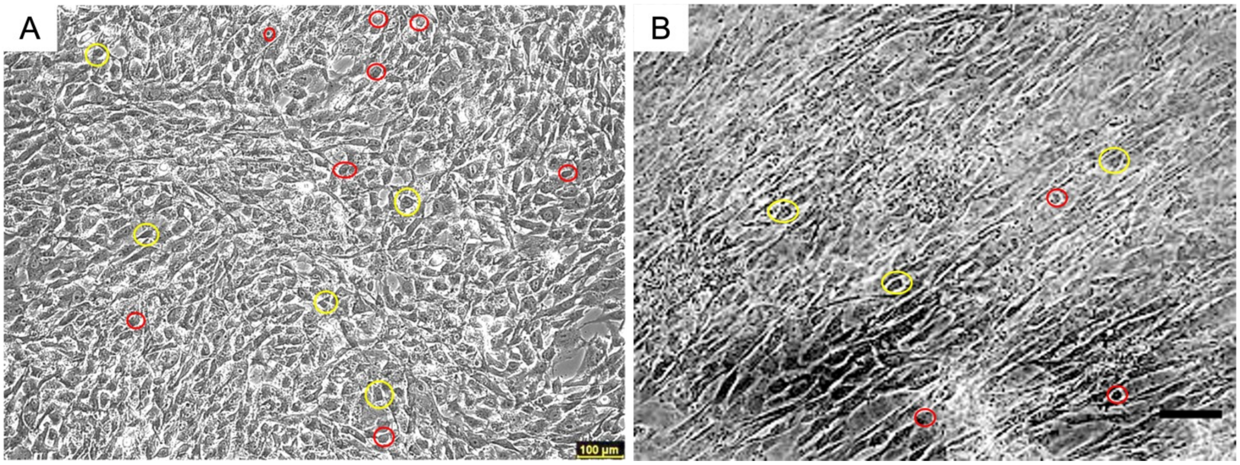

Figure S5. Comparison of the cell populations that do not exhibit the typical bipolar spindle-like morphology, characteristic of cells lying flat in the plane of observation. Representative images of cells cultured on (A) isotropic/control and (B) LC hydrogel samples. Red circles indicate cells with a nearly circular morphology, likely corresponding to cells oriented perpendicular to the plane. Yellow circles highlight cells with a triangular morphology, indicating orientations that are angled relative to the plane, neither fully parallel nor perpendicular.

Figure S6 demonstrates a schematic depiction elucidating the alignment of spins influenced by an external magnetic field.

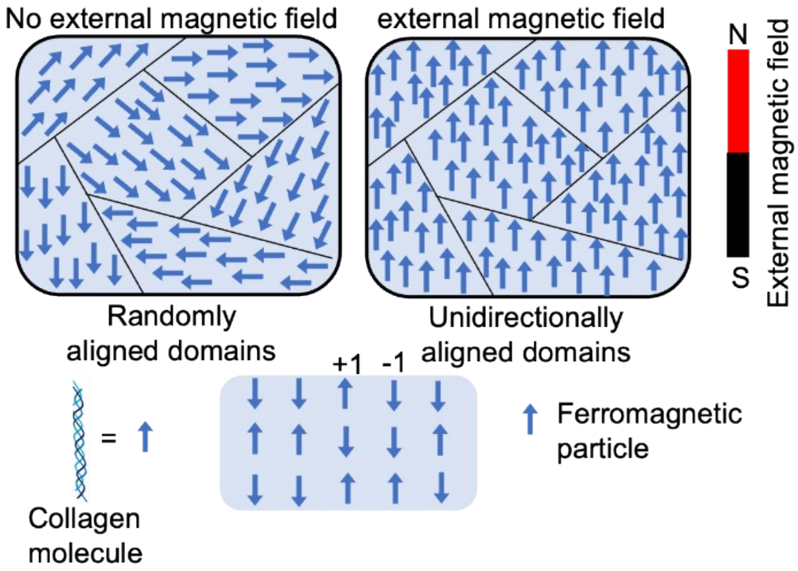

Figure S6. Favorable contributions to the Boltzmann energy distribution in the Monte Carlo Simulation of Ising Theory (applied for the assembly of ferromagnetic particles) occur when spins align in the same direction (+1 or -1). collagen fibrils are treated as analogous entities to spins in the model.
